# Do we neglect nutrition in childhood interstitial lung disease?

**DOI:** 10.1038/s41430-024-01485-x

**Published:** 2024-08-06

**Authors:** Nagehan Emiralioglu, Nural Kiper

**Affiliations:** Department of Pediatric Pulmonology, MD Hacettepe University Faculty of Medicine, Ankara, Turkey

**Keywords:** Diseases, Physics

## Abstract

Growth failure and inadequate weight gain are common problems in childhood interstitial lung diseases (chILD) and these children usually need high calories. It is important to manage both pulmonary functions and nutrition as part of their overall treatment plan and early interventions will help children to improve their quality of life and slow the progression of chronic lung disease. Nutritional evaluation on routine clinical follow-up is important, although there are not any specific guidelines for chILD. Nutritional education, high balanced energy, protein, and fat diet will assist to improve weight gain and maintenance of adequate nutrition status in children with ILD.

## Introduction

Chronic respiratory diseases have important effects on growth and weight gain in children, furthermore growth failure and low weight gain are the most common findings due to increased energy needs in this group. Because of the important effects of malnutrition on pulmonary functions it is necessary to manage both pulmonary functions and nutrition as part of their overall treatment plan [1]. Nutrition also have a critical role in healthy lung development; so weight loss and growth failure are warning signs that clinicians should take attention in this group. Early interventions would help children to improve their quality of life and slow the progression of chronic lung disease. Although there are some clinical trials about the nutrition and chronic respiratory disorders in children, and also guidelines in these patients, there is not enough data in childhood interstitial lung diseases (chILD) [2, 3]. Here, we aimed to take attention about nutritional evaluation of children with interstitial lung diseases.

## Effect of nutrition on chronic respiratory disorders

Nutrition has an important effect in both acute and chronic respiratory disorders. Inadequate nutritional support during the intrauterin period and in early life can compromise the respiratory system functions and result in decline of lung functions, reduced protection against infections, and predisposition to acute and chronic illnesses in childhood [2]. In recent publications, adequate nutrition has been shown in the prevention, development and progression of some chronic diseases like heart disease and malignancy, there is also evidence that nutrition may play a pathophysiological role in the pulmonary health. Nutritional support plays a critical role for better outcomes of chronic respiratory diseases [2, 3].

The prevalence and effect of inadequate caloric intake in children with chILD has not been studied in children. However, there are few recent reports related with malnutrition in idiopathic pulmonary fibrosis (IPF) in adult population. In this report, nutritional indicators predicting malnutrition were low body mass index (BMI), fat free mas index (FFMI) assessed by bioelectrical impedance analysis (BIA), mid-arm circumference, triceps skinfold, serum albumin and transthyretin levels. The prevalence of malnutrition based on BIA (low FFM) was reported 28% in these patients with IPF [4, 5]. BMI evaluation alone underestimates malnutrition in some conditions, so FFMI evaluation may be a good predictive indicator for malnutrition assessment in chronic lung diseases.

Based on these findings, chILD may also cause significant systemic effects, such as weight loss and failure to thrive. These effects would contribute to exercise intolerance, decreased quality of life and increased frequency of pulmonary exacerbations in patients with chILD and adequate nutrition will improve the anthropometric parameters· 6 min walking test, respiratory muscle strength, and health related quality of life that should be evaluated in patients with chILD [6]. Although there is not enough report in chILD, a cohort study consisting 37 children showed that nutritional status had significant effect on disease severity, diffusion capacity, and oxygen saturation of children with ILD [7].

## Nutritional evaluation and growth failure in chILD

Growth failure in children with ILD is related with the increased energy needs, inadequate dietary intake, chronic hypoxia, gastroesophageal reflux and also with the comorbid disorders like cardiac or gastrointestinal system disease and all these conditions should be keep in mind in patients with chronic respiratory disorders [8].

Close monitoring of anthropometric parameters and nutritional status play an important role in the early detection of nutritional deficits. Nutritional evaluation on routine clinical follow-up is important in chILD, however there is not any specific guideline on this issue. Nutritional clinical care in chILD is mostly based on the other chronic respiratory disease guidelines [9].

In every follow-up visit, it is valuable to measure and evaluate weight and height by using standard guidelines, and also it is important to assess BMI and z-scores, FFM, weight for age and height for age z-scores. It should be encouraged to have a well-balanced diet, with high calorically dense food sources. It is also important to evaluate whether these patients have any eating problems and any need to refer supplemental nutrition program.

Although there is not enough data in children, study by Dziekiewicz et al. showed that the prevalence of GERD in children with ILD is as high as 50%, which seems to be higher than healthy pediatric population [10]. Because of the relationship with GERD and microaspirations in the pathogenesis of interstitial lung diseases; it is necessary to evaluate for gastroesophageal reflux disease, swallowing dysfunction in children with interstitial lung disease, regardless of clinical symptoms.

Table 1 summarizes the suggested nutritional status evaluation for patients with chILD.

**Table 1:** Nutritional status evaluation for patients with chILD.

Nutritional care	Initial evaluation	1st month	3rd month	6th month	1st year	Annually
BMI and BMI z score assessment	+	+	+	+	+	
Fat free mass index assessment	+		+	+	+	+
Weight for age assessment	+	+	+	+	+	+
Height for age assessment	+	+	+	+	+	+
Reflux and swallowing evaluation	+				+	
Respiratory muscle strength evaluation	+		+	+	+	+
Caloric intake evaluation	+		+	+	+	+

## Suggestions for nutritional interventions in chILD

Nutritional interventions in addition to the medical treatment are important key factors for the better outcome of patients with chILD. Various dietary components have been shown to reduce pulmonary inflammation and to have beneficial effects in patients with chronic lung diseases. Therefore, nutritional education and behavioral suggestions are recommended for patients with chILD [9].

Nutritional interventions should be applied on a stepwise approach including preventive nutritional counseling, dietary modification, oral nutrition supplements, and enteral tube feeding if necessary. Although there is no clear evidence for the routine use of oral nutrition supplements in ILD it should be considered on an individual basis.

Polymeric enteral tube feeding should be considered when oral interventions have failed to achieve optimum growth and nutritional status [9]. Gastrostomy feeding is usually preferred to nasogastric tubes for long-term nutritional support. Parenteral nutrition should be keep in mind when enteral feeding is unsuccessful due to severe lung disease.

In conclusion, nutrition is an important part of care for children with chILD and better nutritional status is associated with better lung functions and survival. Assessment of anthropometric parameters is a cornerstone of pediatric practice, and nutritional status monitoring plays an important role in the early detection of nutritional problems. Routine nutritional education, including high balanced energy, protein, and fat diet, supplementary foods with additional energy sources, would improve the weight gain and maintenance of adequate nutrition status in children with ILD. Multicentric randomized controlled prospective trials are needed in chILD and nutrition.
